# Cardiac MRI in Infiltrative Disorders: A Concise Review

**DOI:** 10.2174/157340310791162668

**Published:** 2010-05

**Authors:** Neelima Penugonda

**Affiliations:** Department of Internal Medicine, Lankenau Hospital, Wynnewood, PA, USA

**Keywords:** Cardiac MRI, Infiltrative disorders, sarcoidosis, amyloidosis, hemochromatosis.

## Abstract

Cardiac MR imaging is an effective method for noninvasive imaging of the heart. The technology has been limited in the past because of imaging difficulties associated with cardiac motion. In recent years, however, cardiac MR imaging has broadened its spectrum of applications in cardiovascular disease with impressive advances in spatial and temporal resolution and increased imaging speeds. This review presents the current clinical applications of cardiac MR imaging for evaluation of cardiac disease in infiltrative disorders such as amyloidosis, hemochromatosis, and sarcoidosis.

## BACKGROUND

Magnetic resonance imaging (MRI) demonstrates the capability to detect cardiac involvement in various infiltrative disorders at disease presentation and seems to have increased possibilities in comparison to computed tomography (CT) using both multiplanar and multiechoic sequences [1]. Magnetic resonance imaging (MRI) has been shown to be an ideal non-invasive tool for imaging and diagnosing myocardial and pericardial diseases because it combines good spatial resolution, lack of radiation, non-invasiveness, and three-dimensional imaging with highly reproducible measurements. In dilated and hypertrophic cardiomyopathy, MRI is suitable for the diagnosis and quantification of ventricular volume, stroke volume, and myocardial mass. Recent developments in the area of fast imaging techniques and MR contrast agents rapidly are increasing the utility of MRI for studying and assessing myocardial diseases. MRI has become a helpful technique to diagnose and differentiate infiltrative disorders such as amyloidosis, hemochromatosis and sarcoidosis [2].

Our review focuses on the value of cardiac MRI in various forms of myocardial infiltrative diseases.

## PATHOGENESIS OF INFILTRATIVE DISORDERS

### Hemochromatosis

**Fig. (1):**
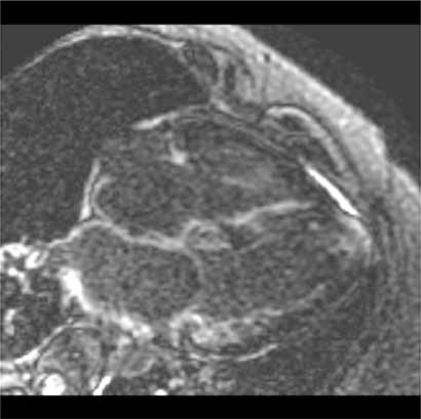
Cardiac MRI Showing Amyloidosis.

The hallmark of hemochromatosis is the deposition of iron in multiple tissue types, most notably the skin, liver, pancreas, thyroid, and heart. Cardiac iron deposition may cause arrhythmias, congestive heart failure and death in patients with primary (Idiopathic) and secondary (acquired) hemochromatosis. Onset of heart failure from iron toxicity generally results in irreversible cardiac damage. Cardiac involvement in primary haemochromatosis is a poor prognostic sign and is the main cause of death in the juvenile form [3]. Usually, the definitive diagnosis is made by subendocardial biopsy in patients with history of primary or secondary hemochromatosis. However, the severity can vary considerable among the various cardiac sites in individual patients which makes subendocardial biopsy unreliable [4]. Non-invasive modalities such as conventional T_2_ -weighted MR sequences can be used routinely to assess the presence of iron deposition in the tissues of patients with hemochromatosis [5] (Fig. **1**). 

T_2_ imaging is highly sensitive in detecting myocardial iron deposition, in patients with moderate-to-severe iron deposition. In these cases T_2_ values are substantially reduced from the normal values of approximately 50 msec or greater to less than 20 msec. When the T_2_ is less than 20 msec, LV systolic function tends to decline progressively, accompanied by an increase in LV end-systolic volume index and LV mass [6]. The cardiac morbidity to some extent is reversible with standard therapies such as phlebotomy or chelation therapy with desferoxamine.

### Amylidosis

**Fig. (2):**
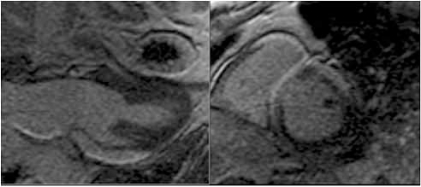
Cardiac MRI Showing Sarcoid Infiltration.

Amyloidosis is characterized by the deposition of glycoproteins in the extracellular space. Cardiac amyloidosis is an interstitial deposition of amyloid fibrils which causes concentric thickening of the atrial and ventricular walls, where it leads to secondary restrictive cardiomyopathy. Involvement of the heart is a common finding and is the most frequent cause of death in amyloidosis [7]. It can be challenging to differentiate amyloidosis from hypertrophic cardiomyopathy, sarcoid and infiltrative lymphoma on echocardiography but easy to differentiate on Cardiac MR. Deposition of amyloid protein causes changes of tissue composition and architecture, and is associated with changes in signal intensity on T1-weighted images. Cardiovascular magnetic resonance imaging should be considered early in the diagnostic work-up of suspected cardiac amyloidosis and is useful in monitoring response to treatment [8]. The characteristic features of cardiac amyloidosis by cardiovascular magnetic resonance imaging - impaired biventricular systolic function, thickened atrioventricular valves, increased atrial septal thickness and left ventricular mass, pleural and pericardial effusions, and the most impressive finding of widespread subendocardial hyperenhancement representing infiltration with amyloid protein.Diffuse heterogenous enchancement of thickened endocardial myocardium on diffuse enchancement on delayed enchancement images along with “Zebra” pattern which is biventricular subendocaridal enchancement leading to a striped appearance (Fig **2**). In noninfiltrative hypertrophic cardiomyopathy, the signal intensity of the myocardium is unchanged compared to that in healthy subjects. T1 quantification of the heart in patients with amyloidosis can also be used to monitor the effect of therapy [9].

### Sarcodosis

**Fig. (3):**
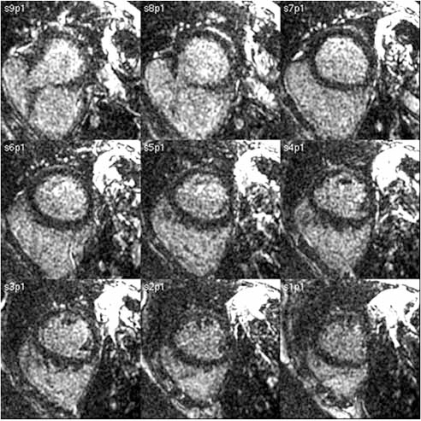
Cardiac MR Images Showing Hemochromatosis.

Sarcoidosis is a granulomatous disease of unknown etiology that can affect any organ. Cardiac involvement is uncommon but has a wide spectrum of clinical manifestations and is potentially fatal [10]. Sudden death accounts for over half of the fatalities from cardiac sarcoidosis but the mechanism has not been established [11]. Cardiac involvement in sarcoidosis produces symptoms in only 5% of patients with sarcoidosis although it has been found in 20–50% of these patients at autopsy. Diagnosis of cardiac sarcoidosis is *via* myocardial biopsy which is limited as there is a possibility of obtaining a false-negative specimen because of patchy cardiac infiltration [12]. During acute myocardial inflammation sarcoid infiltrates are visible on MRI as intramyocardial, epicardial or endocardial hyperenhancement in a non-ischemic pattern with increased signal intensity on both T2-weighted and early gadapentate dimeglumine-enhanced images (Fig. **3**) .The early initiation of corticosteroid therapy prevents malignant arrhythmias and improves left ventricular function as therapy is not effective in the later stages [10].

## DISCUSSION

The initial diagnostic imaging modality employed in assessment of suspected cardiac infiltrative disorders is the readily available transthoracic echocardiography. This is limited in its imaging capability by several well-described factors such as operator experience, restricted field of view, and unfavorable patient body habitus, potentially leading to incomplete assessment of an invading cardiac mass. Transesophageal echocardiography is not limited by issues of suitable acoustic windows but is an invasive technique and has a relatively narrow field of view, thus offering only limited views of relevant structures, in particular the aortic arch, inferior vena cava, and left ventricular apex [13]. Both echocardiographic techniques are limited in their ability to allow characterization of soft-tissue masses.

Pericardial thickness varies in different regions of the heart therefore, its measurement at different levels of the right atrium, right ventricle, and left ventricle is recommended. The pericardium adjacent to the right ventricle can be visualized in up to 100% of individuals whereas the pericardium along the lateral wall of the left ventricle can be visualized in only approximately 61% of cases [14]. Gadolinium-enhanced T1 sequences better delineates the pericardium. Cardiac MR competes with CT as the modality of choice in the recognition of unusual disorders.

The advantages of cardiac MR imaging over the more widely accessible echocardiography are improved resolution and soft-tissue contrast, greater ability to allow tissue characterization, as well as the ability to “phenotype” various forms of cardiomyopathy with high spatial resolution and, through its much larger fields of view, an ability to demonstrate involvement of the adjacent mediastinum and lungs [15]. An added advantage is the reproducible views, which allow accurate comparison between examinations to evaluate any changes in size and appearance. Improved non-invasive surveillance may also aid in the evaluation of new chemotherapeutic agents in treatment of these infiltrative disorders.

Disadvantages include the need for electrocardiographic (ECG) gating, which in the presence of arrhythmia, in particular multiple ectopics, may lead to acquisition artifacts and subsequent image degradation. The multiplanar assessment of the anatomy, tissue characteristics, and functional impact of a cardiac or juxtacardiac mass afforded by cardiac MR imaging should allow early diagnosis thereby avoiding the morbidity associated with late complications.

## CONCLUSIONS

Diagnosing cardiac infiltrative disorders can be challenging but the introduction of cardiac MRI has made early diagnosis possible. As outlined in this article, the strength of MR imaging is that it can provide flow, function, and in some cases metabolic data in a single examination. This is crucial as early treatment improves symptoms and prognosis.
